# Efficacy and safety of tiotropium and olodaterol in COPD: a systematic review and meta-analysis

**DOI:** 10.1186/s12931-017-0683-x

**Published:** 2017-11-25

**Authors:** Marc Miravitlles, Gerard Urrutia, Alexander G. Mathioudakis, Julio Ancochea

**Affiliations:** 1 Pneumology Department, Hospital Universitari Vall d’Hebron., P. Vall d’Hebron 119-129, ES–08035 Barcelona, Spain; 2 Institut d’Investigació Biomèdica Sant Pau (IIB Sant Pau). CIBER de Epidemiología y Salud Pública (CIBERESP), Barcelona, Spain; 3 Division of Infection, Immunity and Respiratory Medicine, University Hospital of South Manchester, The University of Manchester, Manchester, UK; 4 Pneumology Department, Hospital Universitario de La Princesa, Instituto de Investigación Hospital Universitario de la Princesa (IISP) Universidad Autónoma de Madrid, Madrid, Spain

**Keywords:** Systematic review, Metanalysis, COPD, Tiotropium, Olodaterol, Tiotropium/olodaterol fixed dose combination

## Abstract

**Background:**

Long-acting bronchodilators are the cornerstone of pharmacologic treatment of COPD. The new combination of long-acting muscarinic antagonist (LAMA) tiotropium (TIO) and long acting beta-agonists (LABA) olodaterol (OLO) has been introduced as fist line therapy for COPD. This article analyses the evidence of efficacy and safety of the TIO/OLO combination.

**Methods:**

A systematic review and metaanalysis of randomized controlled trials (RCT) with a period of treatment of at least 6 weeks, in patients with COPD confirmed by spirometry, comparing combined treatment with TIO/OLO (approved doses only), with any of the mono-components or any other active comparator administered as an inhalator.

**Results:**

A total of 10 Randomized controlled trials (RCT) were identified (*N* = 10,918). TIO/OLO significantly improved trough FEV_1_ from baseline to week 12 versus TIO, OLO and LABA/ICS (0.06 L, 0.09 L and between 0.04 and 0.05 L, respectively). TIO/OLO improved transitional dyspnea index (TDI) and St. George’s Respiratory Questionnaire (SGRQ) compared with mono-components, with patients more likely to achieve clinically important improvements in TDI (risk ratio [RR]: 1.17, 95% confidence interval [CI]: [1.07, 1.28] versus TIO and RR: 1.14, 95%CI: [1.01, 1.28] versus OLO) and in SGRQ (RR: 1.21, 95%CI: [1.12, 1.30] versus TIO and RR: 1.28, 95%CI: [1.18, 1.40] versus OLO). Patients treated with TIO/OLO showed a significant reduction in the use of rescue medication and no significant differences in frequency of general and serious adverse events were observed between TIO/OLO and mono-components.

**Conclusions:**

Treatment with TIO/OLO provided significant improvements in lung function versus mono-components and LABA/ICS with more patients achieving significant improvements in dyspnea and health status. No differences in adverse events were observed compared with other active treatments.

**Clinical trial registration:**

PROSPERO register of systematic reviews (CRD42016040162).

**Electronic supplementary material:**

The online version of this article (10.1186/s12931-017-0683-x) contains supplementary material, which is available to authorized users.

## Background

Long-acting bronchodilators represent the backbone of available treatments for chronic obstructive pulmonary disease (COPD) [1]. Both long-acting muscarinic antagonists (LAMA) and long acting beta-agonists (LABA) confer significant benefits to patients with COPD, which include but are not limited to improvement in lung function, symptoms, health status and reduction in the exacerbations rate [2–4]. Fixed-dose combinations (FDC) of a LAMA with a LABA were recently introduced and there are increasing number of studies supporting their efficacy and safety [5–7]. LAMA/LABA combination was included in the most recent Global Initiative for Chronic Obstructive Lung Disease (GOLD) strategy as a first line choice therapy for group D (high risk and symptoms) and recommended second option in B (low risk, high symptoms) and C (high risk, low symptoms) groups [1], and the Spanish COPD guidelines recommend the use of LABA/LAMA combinations as first line therapy in patients highly symptomatic and/or at risk for exacerbations [8].

The efficacy and safety of both tiotropium (TIO), the first LAMA introduced in clinical practice, and the LABA olodaterol (OLO) have been extensively evaluated in trials and also in clinical practice [3, 4, 9]. TIO/OLO FDC (5/5 μg), recently approved for the treatment of COPD, has been thoroughly assessed for its efficacy and safety in COPD [10, 11].

Since the combination TIO/OLO is now approved for first line therapy in COPD it is necessary to evaluate the evidence accumulated about its efficacy and safety in these patients. Therefore, the aim of this systematic review was to assess the comparative efficacy, in terms of trough forced expiratory volume in 1 s (trough FEV_1_), quality of life with St. George’s Respiratory Questionnaire (SGRQ), dyspnea (Mahler Transition Dyspnoea Index focal score), exercise capacity, use of rescue medication and safety outcomes of TIO/OLO in combination, either administered in separate or same inhaler, versus the mono-components or any other active comparator (inhaled), in adult patients with COPD.

## Methods

This systematic review methodology is based on a protocol which was registered in PROSPERO register of systematic reviews (CRD42016040162). The report follows the Preferred Reporting Items for Systematic Reviews and Meta-analyses Statement (PRISMA) guidance [12].

### Study selection criteria

Eligible studies were randomized controlled trials (RCT) with a period of treatment and follow up of at least 6 weeks, in patients with a history of COPD confirmed by spirometry, comparing combined treatment with TIO/OLO, either administered in a single or separate inhalers, with any of the mono-components or any other active comparator administered as an inhalator. The RCT should report on at least one of the following outcome measures: trough forced expiratory volume in 1 s (trough FEV_1_), quality of life assessed with the St. George’s Respiratory Questionnaire (SGRQ), symptoms (dyspnea) assessed with the Mahler Transition Dyspnoea Index focal score, exercise capacity, use of rescue medication or safety outcomes, such as all and serious adverse events. The primary objective of this meta-analysis was efficacy.

### Search methods

We searched MEDLINE and EMBASE through OVID, as well as Cochrane CENTRAL (from inception up to May 2016), using appropriate controlled vocabulary and free search terms (detailed search strategy containing the key-words is provided in Additional file 1). We searched trial registries via the World Health Organization International Clinical Trials Platform Search Portal (ICTRP) for ongoing or completed studies (http://apps.who.int/trialsearch/) (accessed in December 2016).

Finally, we checked the reference lists of all trials that were identified by the above mentioned searches.

### Study selection and data extraction

All titles screening as well as full text eligibility assessment were performed by one of the authors (AM). From this list, the references that clearly did not meet the eligibility criteria were excluded. Another reviewer re-assessed and validated study selection (GU). Minor disagreements were resolved through discussion. Data from each study was extracted by one author (AM) and validated by a second author (GU) in detailed tabulated data extraction forms, with a cross check against the original papers. Variables to be extracted from each reference were: i) study identification, ii) methods details, and iii) outcome data (related to those data previously specified in the study selection criteria).When required, additional data were requested to the investigators or the sponsor.

### Risk of bias and heterogeneity assessment

Risk of bias was assessed using the criteria outlined in the Cochrane Handbook for Systematic Reviews of Interventions [13] by one author (AM) and was validated by an additional author (GU). Disagreements were resolved by discussion.

We used the I^2^ statistic to measure heterogeneity among the trials in each analysis [13]. When substantial heterogeneity was identified (I^2^ ≥ 75%.), we reported this and explored possible causes by performing pre-specified subgroup analyses.

### Data analysis

Meta-analyses were performed only when this was meaningful, while the rest of our findings were presented narratively. Dichotomous data were analyzed by calculating risk ratios (RR) and the corresponding 95% confidence intervals (CI), continuous data by calculating mean differences (MD) or standardized mean differences (SMD) and 95% CI and time-to-event data by the inverse variance method.

For data synthesis we used the fixed or random effect models in the absence of important heterogeneity or in the presence of moderate heterogeneity, respectively. In case of substantial heterogeneity, which cannot be resolved by performing the subgroup analyses, we did not perform meta-analysis. Review Manager 5.3 software was used for this meta-analysis.

## Results

**Fig. 1:**
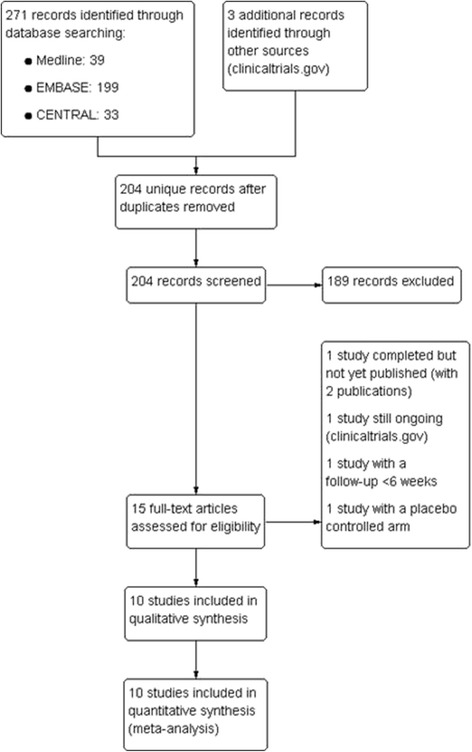
Flow diagram of study selection

Search strategies yielded 201 unique references, of which five met the eligibility criteria. In addition, reports of two more unpublished studies were identified in ICTRP (Fig. 1: Flow diagram of study selection). We identified a total of 10 RCT involving 10,918 participants that were eligible for the review: ANHELTO 1 & 2 (published in one article) [14], ENERGITO [15], MORACTO 1 & 2 [16, 17], OTEMTO 1 & 2 [18, 19], TONADO 1 & 2 [20, 21] and VIVACITO [22]. We identified one additional RCT that has been completed published only as a protocol [23, 24] (PHYSACTO), and one more trial that is still ongoing (DYNAGITO) [25].

Another two RCT assessing the combined treatment with TIO/OLO were excluded due to the short follow up period (4 weeks) [26], or the lack of a comparison with any of the mono-components or an active control (TORRACTO) [27].

### Description of the studies

Details on the characteristics of the included studies are provided in Table 1 (Characteristics of the included studies: summary) and Additional file 2: Table S1.

**Table 1:** Characteristics of the included studies: summary

Study, Year	Intervention (Participants)	Design, *Follow up*	Multicenter/ Double blind	Baseline characteristics	Risk of Bias
ANHELTO 1 & 2 2014	Tiotropium 18 μg & Olodaterol 5 μg (1135) Tiotropium 18 μg & Placebo (1136) Administered in two separate inhalers (Respimat® inhaler for Olodaterol and Spiriva Handihaler® for Tiotropium), once-daily × 12 wk	Parallel RCT, ***15*** *weeks*	✓/✓	Mean(SD) age – 51.7(9) Male – 51.7% Ex-smokers – 51% GOLD 2–58.9%, GOLD 3–41.1%	Low
ENERGITO 2016	Tiotropium 2.5 μg & Olodaterol 5 μg Tiotropium 5 μg & Olodaterol 5 μg [FDC via Respimat®, once-daily × 6 wk] Salmeterol & Fluticasone 50/250 μg Salmeterol & Fluticasone 50/500 μg (*N* = 229 × 4)	Crossover RCT, Participants completed 4/4 arms ***>6*** *weeks*	✓/✓	Mean(SD) age - 63.6(7.6) Male – 64.6% Ex-smokers – 55.5% GOLD 2–72.1%, GOLD 3–27.9%	Low
MORACTO 1 & 2 2015	Tiotropium 2.5 μg & Olodaterol 5 μg Tiotropium 5 μg & Olodaterol 5 μg [FDC via Respimat®, once-daily × 6 wk] Tiotropium 5 μg Olodaterol 5 μg Placebo (*N* = 586 × 4)	Crossover RCT, Participants completed 4/5 arms, ***6*** *weeks*	✓/✓	Mean(SD) age – 61.7(7.7) Male – 71.2% Ex-smokers – 60.9% GOLD 2–71%, GOLD 3–28%	Low
OTEMTO 1 & 2 2015	Tiotropium 2.5 μg & Olodaterol 5 μg (404) Tiotropium 5 μg & Olodaterol 5 μg (406) [FDC via Respimat®, once-daily × 12 wk] Tiotropium 5 μg (407) Placebo (406)	Parallel RCT, ***15*** *weeks*	✓/✓	Mean(SD) age – 64.7(8.4) Male – 60.9% Ex-smokers – 52.9% GOLD 2–64.4%, GOLD 3–35.6%	Low
TONADO 1 & 2 2015	Tiotropium 2.5 μg & Olodaterol 5 μg (1030) Tiotropium 5 μg & Olodaterol 5 μg (1029) [FDC via Respimat®, once-daily × 52 wk] Tiotropium 2.5 μg (1032) Tiotropium 5 μg (1033) Olodaterol 5 μg (1038)	Parallel RCT, ***52*** *weeks*	✓/✓	Mean(SD) age - 646(8.3) Male – 73.3% Ex-smokers – 63.% GOLD 2–50.1%, GOLD 3,4–49.9%	Low
VIVACITO 2015	Tiotropium 2.5 μg & Olodaterol 5 μg Tiotropium 5 μg & Olodaterol 5 μg [FDC via Respimat®, once-daily × 6 wk] Tiotropium 2.5 μg Tiotropium 5 μg Olodaterol 5 μg Placebo (*N* = 259 × 6)	Crossover RCT, Participants completed 4/6 arms, ***6*** *weeks*	✓/✓	Mean(SD) age - 61.1(7.7) Male - 58.9% Ex-smokers - 37.4% GOLD 2–63.5%, GOLD 3,4–36.5%	Low

Six studies had a parallel group design, with a sample size ranging from 607 and 1577 participants, whereas the other four had a crossover design that included between 219 and 295 participants (mean 1091; median 809). All studies except ANHELTO 1 & 2 (which were conducted exclusively in the US) were multinational with a wide range of participating countries. Treatment duration was 6 weeks (4 studies), 12 weeks (4 studies) and 24 weeks (2 studies), with an additional extension up to 54 weeks in the later ones.

All trials (except ANHELTO 1 & 2) assessed once-daily TIO/OLO FDC, which was administered with the use of a single inhaler (Respimat®). By contrast, in ANHELTO 1&2, the combination treatment TIO + OLO was administered using two different inhalers (HandiHaler® and Respimat®). Regarding the dosing of TIO in the combined treatment, ANHELTO 1 & 2 used TIO 18 μg, whereas all the rest assessed two different FDC using high and low doses of TIO (5 μg and 2.5 μg). OLO was always administered at the same dose of 5 μg. For the purposes of this review, only data from TIO/OLO FDC arms where high dose of TIO (5 μg) were used have been included, as the low dose (2.5 μg) is not marketed.

Nine studies had a control group with TIO (5 or 18 μg), and five a control group with OLO (5 μg). Only one study compared the combined therapy with TIO/OLO versus the combined therapy with salmeterol plus fluticasone at two different dose combination (50/500 μg or 50/250 μg) [15].

Overall, the inclusion criteria and populations’ characteristics of the studies were very homogeneous. Participants were aged ≥40 years, current or ex-smokers with a smoking history of more than 10 pack-years, mostly with moderate to severe COPD; only TONADO 1 & 2 included participants with very severe disease (FEV_1_ < 30% predicted, 10.8% of the participants). All studies required participants to be able to inhale medication in a competent manner from the Respimat® or HandiHaler® inhalers as well as to perform technically acceptable pulmonary function tests, and maintain records (paper diary) as required.

### Risk of bias of the included studies

Risk of bias was deemed low for all domains evaluated in all included trials (details in Additional file 3: Figure S1 and Additional file 4: Figure S2). Risk of bias was assessed according the criteria outlined in the Cochrane Handbook. Where no sufficient details were provided in the article (i.e. allocation concealment), these were requested to the sponsor who provided further details.

### Efficacy of the intervention

**Fig. 2:**
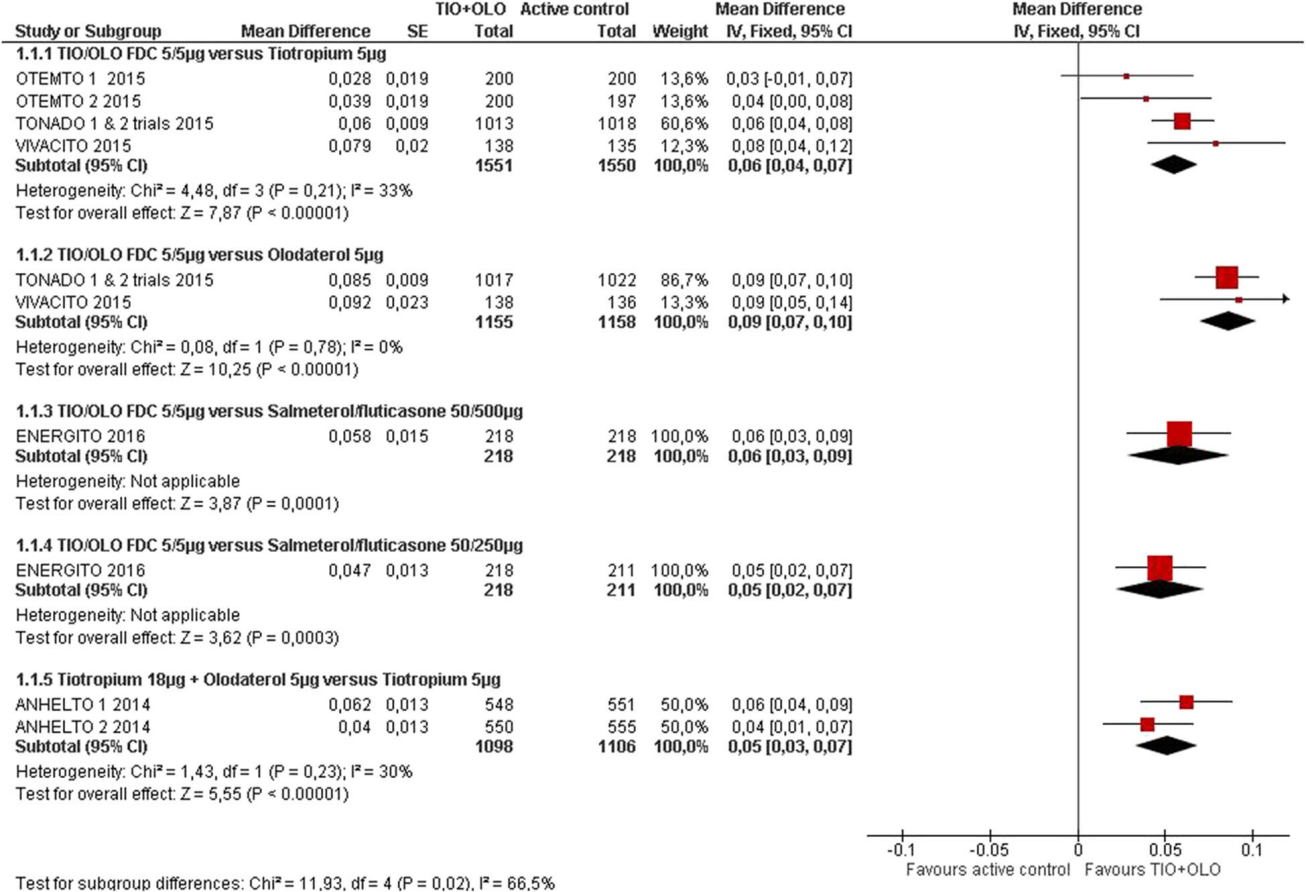
Trough FEV_1_

Trough FEV_1_ was reported in eight of the RCT [14, 15, 18–22]. Overall, the combined therapy proved to be superior to the mono-components in all studies (Fig. 2: Trough FEV_1_). TIO/OLO was associated with significantly higher trough FEV_1_ when compared with TIO (MD 0.06 [0.04 to 0.07], I^2^ = 33%) (5 RCT with 3101 patients) or OLO (MD 0.09 [0.07–0.10], I^2^ = 0%) (3 RCT with 2313 patients). TIO/OLO showed a statistically significant greater improvement in trough FEV_1_ after 6 weeks of treatment compared to both doses of salmeterol plus fluticasone (with an improvement ranging between 42 and 58 mL). When treatment was administered in separate inhalers, TIO + OLO (18/5 μg) resulted in significant improvements over TIO (18 μg) in trough FEV_1_ (treatment differences: 62 mL [*P* < 0.001] in ANHELTO 1; 40 mL [*P* = 0.0029] in ANHELTO 2) [14].

**Fig. 3:**
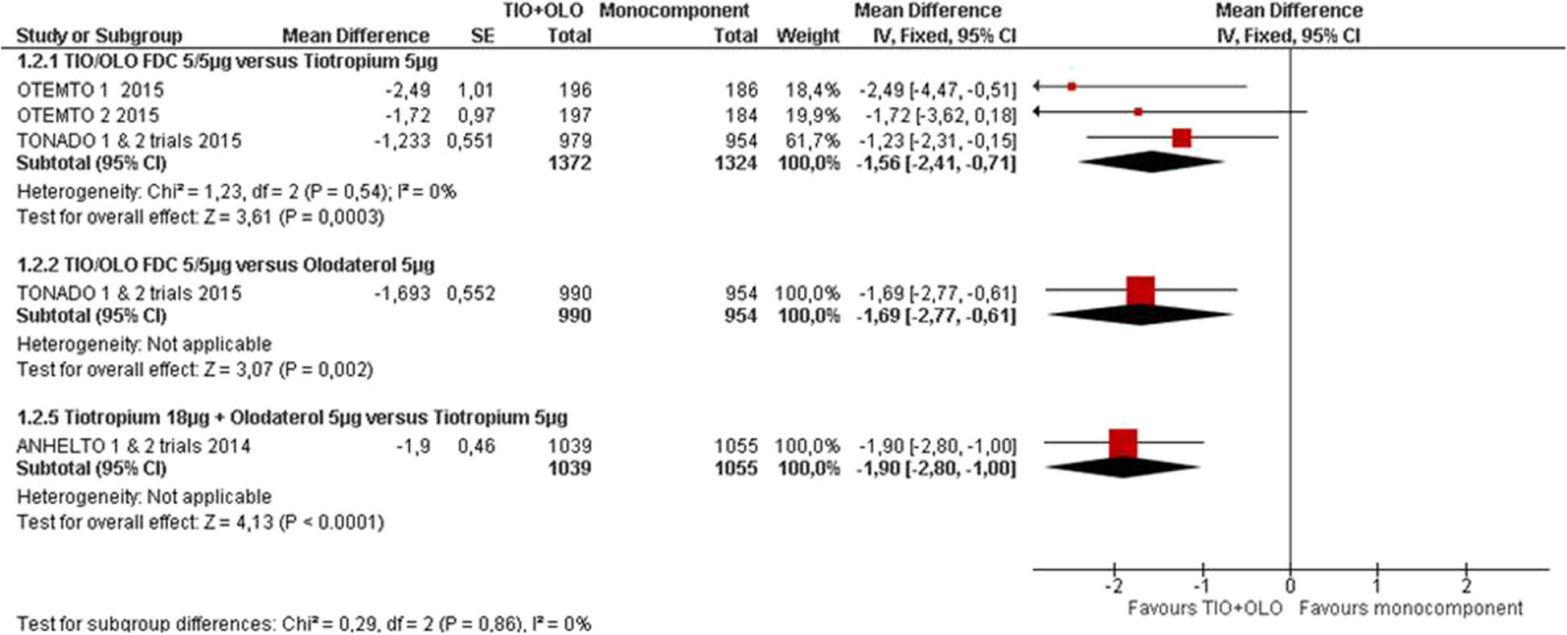
Quality of life SGRQ: change from baseline

**Fig. 4:**
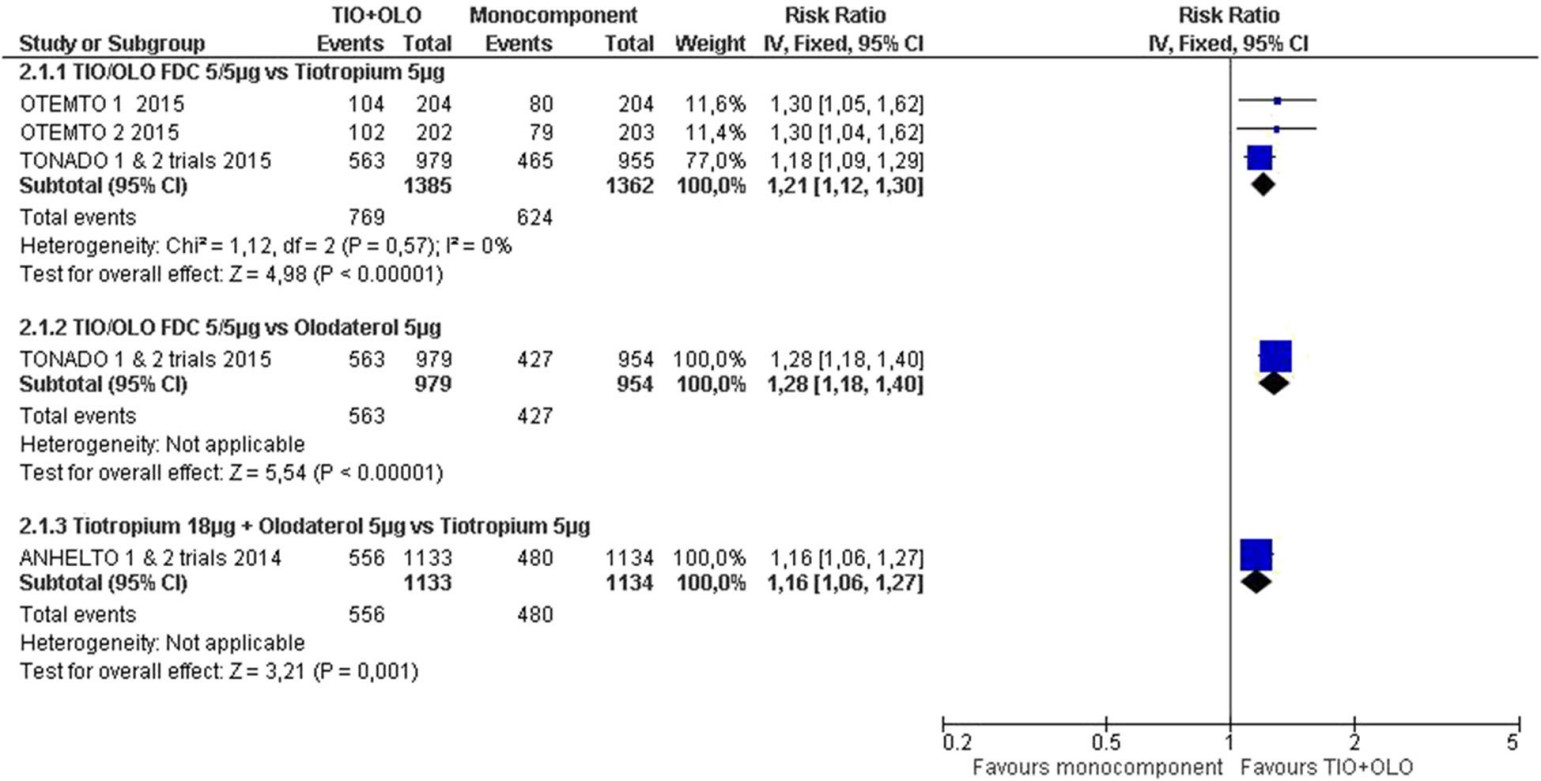
Quality of life SGRQ: responders

TIO/OLO was associated with an improved quality of life compared to TIO (MD -1.56 [−2.41 to −0.71], I^2^ = 0%) in 4 RCT with 2697 participants [14, 18, 19] or OLO (−1.69 [−2.77 to −0.61]) in 2 RCT with 1933 participants [20, 21] (Fig. 3: Quality of life SGRQ: change from baseline). More participants receiving TIO/OLO had a clinically meaningful difference in SGRQ compared to TIO [14, 18, 19] (RR 1.21 [1.12 to 1.30], I^2^ = 0%) or OLO [20, 21] (RR 1.28 [1.18 to 1.40]) (Fig. 4: Quality of life SGRQ: responders).

Similar results were found for TIO + OLO (18/5 μg) vs TIO, both in the SGRQ total score change [14] (MD: -1.90 [−2.80 to −1.00]) and SGRQ responders rate (RR 1.16 [1.06 to 1.27]) (Figs. 3 and 4).

**Fig. 5:**
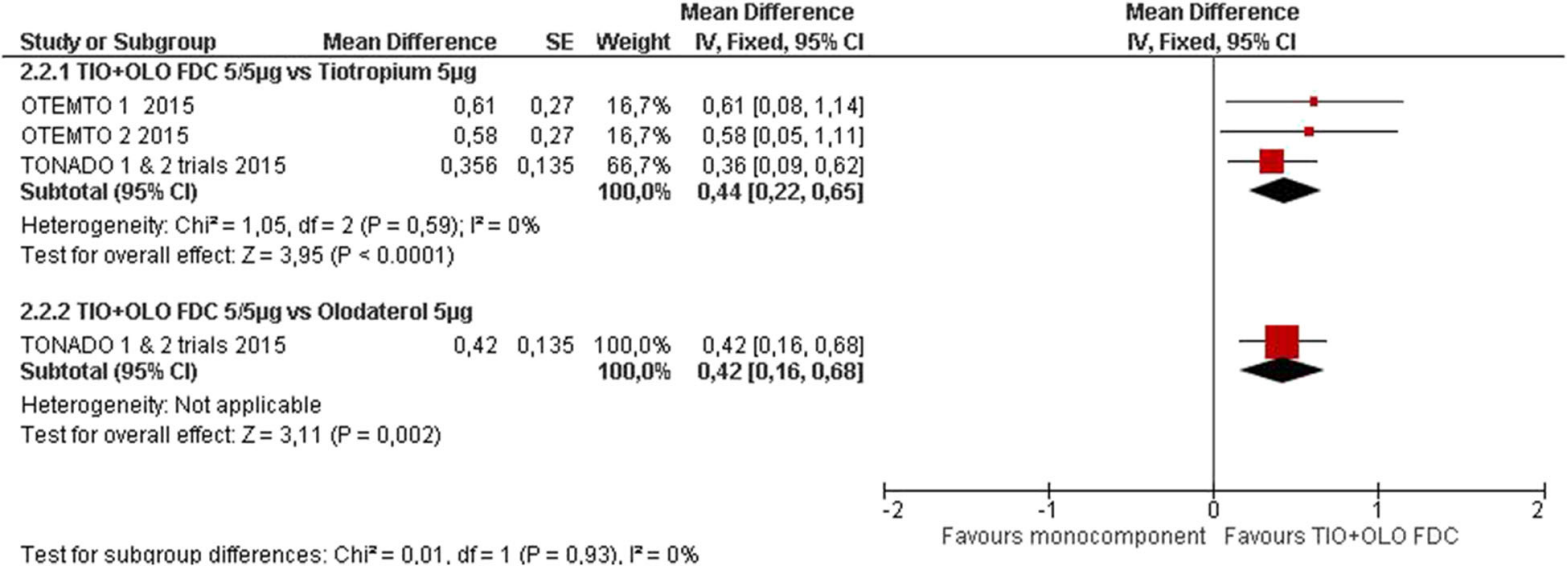
Symptoms TDI: change from baseline

**Fig. 6:**
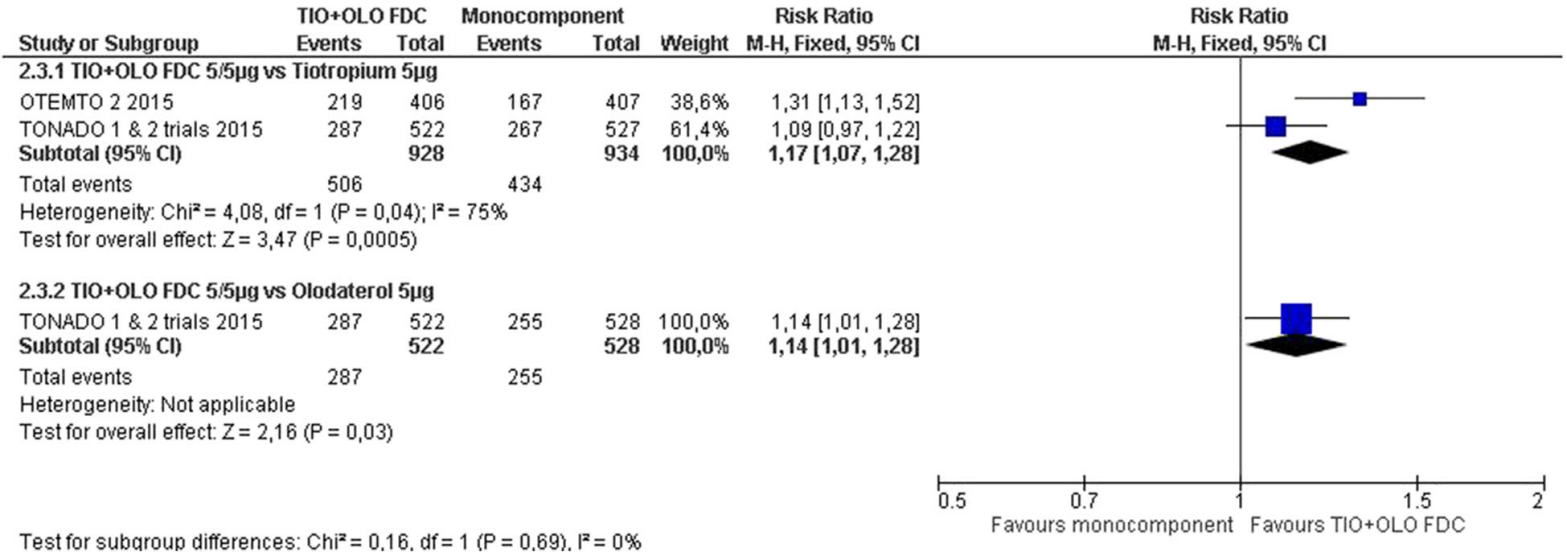
Symptoms TDI: responders

Four studies measured the Mahler Transition Dyspnea Index (TDI) [18–21]. TIO/OLO led to improved TDI compared to TIO (MD 0.43 [0.22 to 0.65], I^2^ = 1%) and OLO (RR 0.42 [0.16 to 0.68]) (Fig. 5: Symptoms TDI: change from baseline). More participants receiving TIO/OLO had a clinically meaningful difference in TDI score (≥1.0 unit) compared to TIO (RR 1.17 [1.07 to 1.28], I2 = 75%) or OLO (RR 1.14 [1.01 to 1.28]) (Fig. 6: Symptoms TDI: responders).

Rescue medication usage was lower with TIO + OLO (18/5 μg) than with TIO [14, 18–21]. On average, TIO + OLO reduced the number of days using rescue medication by 8.5 days (95% CI 4.2, 12.8) in ANHELTO 1 and by 7.2 days (95% CI 3 to 11.49) in ANHELTO 2. In OTEMTO 1 & 2, the use of rescue medication over 24 h was lower in patients receiving TIO/OLO compared to TIO after 12 weeks (*P* < 0.05) (post-hoc analysis). In TONADO 1 & 2, TIO/OLO provided reductions in adjusted weekly mean daily (24-h) rescue medication use compared to the mono-components throughout the 52-week treatment period.

MORACTO 1 & 2 trials showed a trend over increased endurance time for TIO/OLO compared to TIO (MD 8.06 [−13.76 to 29.87], I^2^ = 99%) or OLO (MD 23.67 [−21.34 to 68.69], I^2^ = 100%); however, these results are limited by the significant heterogeneity between the two included trials.

**Fig. 7:**
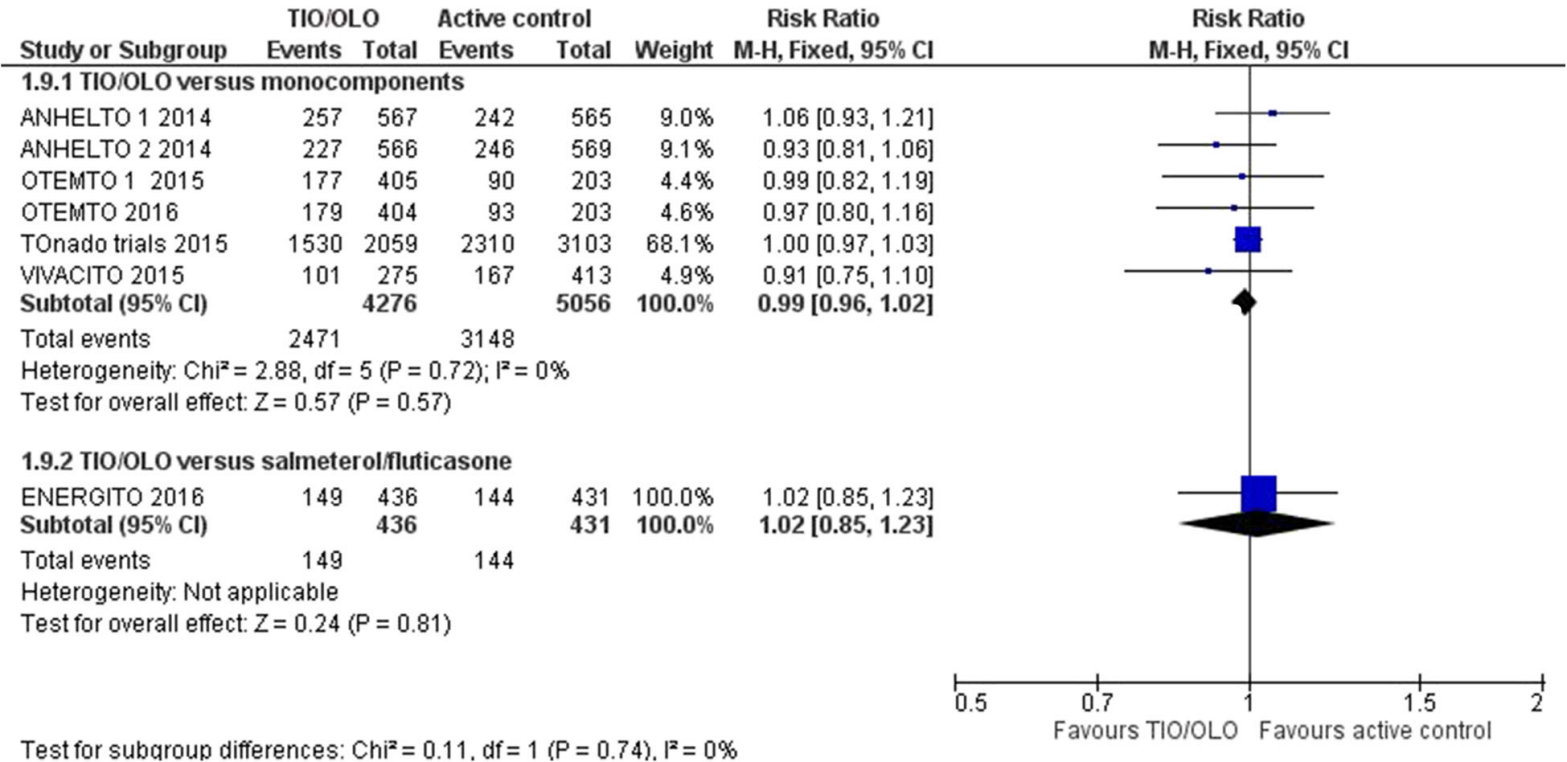
All adverse events

Regarding safety, no differences were observed in the frequency of general and serious adverse events between TIO/OLO and the mono-components. All adverse events were reported in ANHELTO 1 & 2, OTEMTO 1 & 2, TONADO 1 & 2, VIVACITO and ENERGITO trials. No significant differences between groups were observed when comparing TIO/OLO versus mono-components (RR 0.99 [0.96 to 1.02], I2 = 0%) or versus salmeterol fluticasone (RR 1.02 [0.85 to 1.23]) (Fig. 7: All adverse events). Serious adverse events were assessed in ANHELTO 1 & 2, OTEMTO 1 & 2, TONADO 1 & 2, VIVACITO and ENERGITO trials. A similar between group distribution was observed between participants receiving TIO/OLO versus mono-components or placebo (RR 0.99 [0.88 to 1.11], I2 = 49%). Also, ENERGITO trial concluded similar number of serious adverse events were observed between participants receiving TIO/OLO versus fluticasone/salmeterol (RR 0.80 [0.39 to 1.65]).

## Discussion

The results of our analysis showed that TIO/OLO is an effective and safe treatment for patients with COPD of a very wide range of severity. The data obtained in the RCT have demonstrated significant improvements in through FEV1, SGRQ, dyspnea scores and a reduction in the use of rescue medication compared with the mono-components, and a significant improvement in through FEV1 in one study compared to LABA/ICS combination. No safety issues were identified in the comparative analysis with the mono-components.

These results concur with those obtained with other LABA/LAMA FDC, that also demonstrate to provide better outcomes compared with mono-components [28–30] and with LABA/ICS [31, 32] and support the recent GOLD update that recommends LABA/LAMA as first line therapy in patients classified as GOLD D (more symptoms and increased risk of exacerbations) [1]. In a different approach based on clinical phenotypes, the Spanish guideline of management of COPD in its 2017 update recommends LABA/LAMA as first line therapy in patients classified as high risk (either FEV_1_ (% predicted) < 50% or MRC ≥ 2 or >1 exacerbations or 1 hospitalization in the previous year) irrespective of the phenotype, with the exception of the asthma-COPD overlap (ACO) in which LABA/ICS is considered the preferred initial option [8].

The current analysis was based on 10 completed RCTs with TIO/OLO in COPD patients. Of these, only 2 RCTs used the combination of TIO and OLO administered with separate inhalers [14]. The remaining 8 RCTs used TIO/OLO FDC administered with the same inhaler (Respimat®). These studies evaluated adult patients (≥ 40 years), predominantly men, smokers or ex-smokers, with moderate and severe COPD, even two of the trials included up to 10.8% of patients with very severe COPD (FEV_1_ (%) < 30%). All trials were considered of high-quality, both in terms of design and execution, and the risk of bias in the estimation of the effect was perceived as low.

The combination of TIO/OLO demonstrated a mean improvement in trough FEV_1_ of between 60 mL to 90 mL over TIO and OLO, respectively. This improvement is in line of the mean improvement observed with dual bronchodilators versus LAMA alone [5]. Interestingly, the superiority of TIO/OLO in terms of lung function was observed consistently in all analyzed RCTs.

The results observed in lung function were paralleled with significant improvements in the SGRQ. The mean differences in scores did not reach the 4 units considered clinically relevant [33], but the probability of having a response superior to 4 units were significantly increased by 21% versus TIO and 28% versus OLO. Mean differences of around 4 units have only been observed between dual bronchodilation and placebo [33], but studies comparing LABA/LAMA combinations with mono-components have shown mean differences below that threshold, as observed in our analysis [28, 30]. However it is important to highlight the increased likelihood of achieving a clinically significant improvement in quality of life with TIO/OLO, which may be very relevant in more severe patients.

The current analysis has shown that TIO/OLO led to improved TDI compared to TIO and OLO alone. Patients on TIO/OLO had 17% higher probability to experience an improvement >1 unit in the TDI dyspnea score versus patients treated with TIO and 14% versus OLO. These results are also consistent with those reported for other LABA/LAMA FDC, where combinations improve significantly TDI scores over monotherapies, but mean differences do not achieve the 1 unit threshold [28, 30]. However, all combinations increase the probability of a patient to reach a clinically significant improvement in dyspnea.

Rescue medication use and/or days free of rescue medication were assessed in six trials. TIO/OLO significantly reduced the use of rescue medication. This effect was observed throughout the 52-week follow-up in TONADO 1 & 2. The decrease in the use of rescue medication is one of the proposed markers for clinical control in COPD [34] and is associated with a decrease risk of exacerbations [35].

In two RCTs, TIO/OLO showed a non-significant trend towards an increase in exercise capacity compared to both monotherapies. These results are similar to those obtained with other LABA/LAMA FDC [36] and indicate that exercise limitation in COPD is multifactorial and significant improvements in lung function do not immediately translate into significant increases in exercise capacity. Other factors such as comorbidities and deconditioning may influence the reduced exercise capacity in COPD.

Regarding safety, no differences were observed in the frequency of general and serious adverse effects between TIO/OLO FDC and the mono-components; thus reassuring the excellent safety profile of this combination [37].

The strengths of the review are those typical of a systematic review: exhaustive search for studies, a reasonably high number of available studies, studies of high methodological quality, possibility of performing a meta-analysis, low heterogeneity and high consistency between studies. However, as limitations we highlight that all clinical trials were performed by the same pharmaceutical company, common limitation when a drug is reviewed by a meta-analysis. Another limitation is that it was not possible to do a subgroup analysis according to the patient’s baseline level of severity and a different design related to variable duration of clinical trials (between 6 and 24 weeks).

## Conclusions

TIO/OLO is an effective and safe treatment for patients with COPD of any degree of severity. The improvements obtained in lung function are superior to those observed with monotherapies or with LABA/ICS combination. These improvements also translate with different intensity to improvements in other patient-reported outcomes.

## Additional files

Additional file 1: Figure S3. Detailed search strategy. (DOCX 14 kb)

Additional file 2: Table S1. Characteristics of the included studies. (DOCX 121 kb)

Additional file 3: Figure S1. Risk of bias graph. (JPEG 40 kb)

Additional file 4: Figure S2. Risk of bias summary. (JPEG 60 kb)
