# Supplementary material for: Efficacy and safety of tiotropium and olodaterol in COPD: a systematic review and meta-analysis
Source: Respir Res. 2017 Nov 25;18:196. doi: 10.1186/s12931-017-0683-x (PMC5702233; doi:10.1186/s12931-017-0683-x)
Supplement: Supplementary file 1 — Detailed search strategy. (DOCX 14 kb) [file 12931_2017_683_MOESM1_ESM.docx]

**Search strategy**

We used either terms describing the combination of tiotropium with olodaterol, such as stiolto and spiolto or the combination of the keywords tiotropium and olodaterol as well as alternative names (detailed in search strategy) for these substances.

We have not used filters for randomized trials but we excluded animal studies.

Database: Ovid MEDLINE(R) In-Process & Other Non-Indexed Citations, Ovid MEDLINE(R) Daily and Ovid MEDLINE(R) <1946 to Present>

Search Strategy:

--------------------------------------------------------------------------------

1 Olodaterol.tw. (75)

2 (BI 1744 CL or UNII-65R445W3V9 or 65R445W3V9 or DB09080 or D10020).tw. (5)

3 1 or 2 (77)

4 exp Tiotropium Bromide/ (831)

5 tiotropium.tw. (1134)

6 4 or 5 (1280)

7 3 and 6 (40)

8 stiolto.tw. (3)

9 spiolto.tw. (0)

10 7 or 8 or 9 (40)

11 exp animals/ (20069630)

12 exp humans/ (15844815)

13 11 not 12 (4224815)

14 10 not 13 (39)

Database: Embase <1980 to 2016 Week 12>

Search Strategy:

--------------------------------------------------------------------------------

1 exp Olodaterol/ (258)

2 exp Olodaterol Respimat/ (258)

3 exp Olodaterol Hydrochloride/ (258)

4 Olodaterol.tw. (185)

5 (BI 1744 CL or BI-1744 CL or UNII-65R445W3V9 or CHEBI:83309 or 65R445W3V9 or DB09080 or D10020).tw. (72)

6 1 or 2 or 3 or 4 or 5 (333)

7 exp Tiotropium Bromide/ (4367)

8 tiotropium.tw. (2262)

9 (BA 679 BR or BA-679 BR or BA679 BR or BR, BA 679 or 679 BR, BA or 136310-93-5 or BA 679BR).tw. (24)

10 7 or 8 or 9 (4543)

11 6 and 10 (187)

12 exp olodaterol plus tiotropium/ (49)

13 exp olodaterol plus tiotropium bromide/ (49)

14 exp olodaterol hydrochloride plus tiotropium bromide/ (49)

15 Stiolto.tw. (4)

16 Spiolto.tw. (0)

17 11 or 12 or 13 or 14 or 15 or 16 (202)

18 exp animals/ not exp humans/ (4105680)

19 17 not 18 (199)
