# Supplementary material for: Efficacy and safety of tiotropium and olodaterol in COPD: a systematic review and meta-analysis
Source: Respir Res. 2017 Nov 25;18:196. doi: 10.1186/s12931-017-0683-x (PMC5702233; doi:10.1186/s12931-017-0683-x)
Supplement: Supplementary file 2 — Characteristics of the included studies. (DOCX 121 kb) [file 12931_2017_683_MOESM2_ESM.docx]

**e-supplement.**

**Title:** Efficacy and safety of Tiotropium and Olodaterol: A Systematic Review and Meta-Analysis.

**Authors:** 1. Marc Miravitlles, 2. G Urrutia, 3. Alexander G Mathioudakis, 4. Julio Ancochea.

**Centre:** 1. Pneumology Department, Hospital Universitari Vall d’Hebron. CIBER de Enfermedades Respiratorias (CIBERES). Barcelona, Spain. 2. 4. Pneumology Department, Hospital Universitario de La Princesa, Instituto de Investigación Hospital Universitario de la Princesa (IISP) Universidad Autónoma de Madrid, Madrid.

**Table e1.** Search strategy.

Database: Ovid **MEDLINE**(R) In-Process & Other Non-Indexed Citations, Ovid **MEDLINE**(R) Daily and Ovid **MEDLINE**(R) <1946 to Present>

Search Strategy:

--------------------------------------------------------------------------------

1 Olodaterol.tw. (75)

2 (BI 1744 CL or UNII-65R445W3V9 or 65R445W3V9 or DB09080 or D10020).tw. (5)

3 1 or 2 (77)

4 exp Tiotropium Bromide/ (831)

5 tiotropium.tw. (1134)

6 4 or 5 (1280)

7 3 and 6 (40)

8 stiolto.tw. (3)

9 spiolto.tw. (0)

10 7 or 8 or 9 (40)

11 exp animals/ (20069630)

12 exp humans/ (15844815)

13 11 not 12 (4224815)

14 10 not 13 (39)

Database: **Embase** <1980 to 2016 Week 12>

Search Strategy:

--------------------------------------------------------------------------------

1 exp Olodaterol/ (258)

2 exp Olodaterol Respimat/ (258)

3 exp Olodaterol Hydrochloride/ (258)

4 Olodaterol.tw. (185)

5 (BI 1744 CL or BI-1744 CL or UNII-65R445W3V9 or CHEBI:83309 or 65R445W3V9 or DB09080 or D10020).tw. (72)

6 1 or 2 or 3 or 4 or 5 (333)

7 exp Tiotropium Bromide/ (4367)

8 tiotropium.tw. (2262)

9 (BA 679 BR or BA-679 BR or BA679 BR or BR, BA 679 or 679 BR, BA or 136310-93-5 or BA 679BR).tw. (24)

10 7 or 8 or 9 (4543)

11 6 and 10 (187)

12 exp olodaterol plus tiotropium/ (49)

13 exp olodaterol plus tiotropium bromide/ (49)

14 exp olodaterol hydrochloride plus tiotropium bromide/ (49)

15 Stiolto.tw. (4)

16 Spiolto.tw. (0)

17 11 or 12 or 13 or 14 or 15 or 16 (202)

18 exp animals/ not exp humans/ (4105680)

19 17 not 18 (199)

**Table e2.** Characteristics of the included studies.

| **ANHELTO 1 & 2** (NCT01696058 and NCT01694771) | |
| --- | --- |
| Objective | To assess efficacy and safety of 12 weeks, once daily, orally inhaled co-administration of olodaterol 5 μg (delivered by the Respimat® Inhaler) and tiotropium (delivered by the Handihaler® as Spiriva Handihaler®), compared to tiotropium (Spiriva Handihaler®) monotherapy on lung function in patients with COPD |
| Methods | Parallel design (two arms)  Double-blind  Phase III  Multicentre (90 & 98 centres)  Country: USA  Follow up (& end point): 12 weeks  Run-in period: 2 weeks |
| Participants  (elegibility) | Inclusion criteria:   - Diagnosis of COPD and with the following spirometric criteria: relatively stable airway obstruction with a post-bronchodilator FEV_1_ ≥30 % and <80% of predicted normal and post-bronchodilator FEV_1_/FVC <70% (GOLD 2–3) - Age ≥40 years - Current or ex-smokers with a smoking history of more than 10 pack years - To be able to perform technically acceptable pulmonary function tests, and maintain records (paper diary) - To be able to inhale medication in a competent manner from the Respimat Inhaler as well as the Handihaler   Key exclusion criteria:   - History of asthma - Significant disease other than COPD (thyrotoxicosis, paroxysmal tachycardia, myocardial infarction within 1 year, unstable or life-threatening cardiac arrhythmia) - Hospitalization for heart failure within the past year - Clinically relevant abnormal baseline haematology, blood chemistry, or urinalysis |
| Participants  (characteristics) | **ANHELTO 1**  Age (mean, SD): 64.3 - 64.8 (9.1)  Gender (male): 49.2 - 50.4%  COPD diagnosis, mean (SD), years: 7.9 – 8.5 (6.1 – 7.5)  GOLD: 2 (59.8 - 60.5%), 3 (39.3 – 40.2%), 4 (0 - 0.2%)  COPD diagnosis: 7.9 – 8.5 years  % of predicted normal FEV_1_: 53.9 – 54.2% (13.0 – 13.0)  Any pulmonary medication at baseline: 70.4 – 71.9%  Current smokers: 49.7 – 52.2%  Smoking history (pack-years): 49.7 – 52.2  **ANHELTO 2**  Age (mean, SD): 63.6 – 64.6 (8.9 – 9.0)  Gender (male): 53.3 – 53.9%  COPD diagnosis, mean (SD), years: 7.1 – 8.2 (6.3 – 7.2)  GOLD: 2 (55.7 - 59.7%), 3 (40.1 – 44.3%), 4 (0 - 0.2%)  COPD diagnosis: 7.1 – 8.2 years  % of predicted normal FEV_1_: 53.0 – 53.6% (13.6 – 13.9)  Any pulmonary medication at baseline: 70.5 – 74.4%  Current smokers: 45.8 – 48.2%  Smoking history (pack-years): 26.8 – 28.1 |
| Participants  (flow) | **ANHELTO 1**  Screened: 1813  Randomised: 1134   - Tiotropium + olodaterol: 567 - Tiotropium + placebo: 567   Treated: 99.82% (1132/1134)  Completed: 92.93% (1052/1132)  **ANHELTO 2**  Screened: 1964  Randomised: 1137   - Tiotropium + olodaterol: 568 - Tiotropium + placebo: 569   Treated: 99.82% (1135/1137)  Completed: 92.43% (1051/1137) |
| Interventions | Experimental:   - Tiotropium 18 μg, one capsule of tiotropium dry powder (via the HandiHaler^®^) and olodaterol 5 μg, Respimat^®^ inhaler, once daily   Control:   - Tiotropium 18 μg, one capsule of tiotropium dry powder (via the HandiHaler^®^) and Placebo, Respimat^®^ inhaler, once daily   Treatment duration: 12 weeks  Co-interventions:  Inhaled corticosteroids, oral (≤10 mg prednisone per day, or equivalent) and injected steroids, cromolyn sodium/nedocromil sodium, antihistamines, anti leukotrienes, methylxanthines, mucolytic, and theophyllines were allowed  Albuterol was provided as rescue medication only |
| Outcomes | Primary:   - FEV_1_ AUC_0–3_ - FEV_1_ responses (i.e., change from baseline)   Secondary:   - St George’s Respiratory Questionnaire total score - Peak FEV_1_ - FVC AUC_0–3_ - Peak and trough FVC responses - Rescue medication use over the 12-week treatment period - Adverse events, serious adverse events, vital signs, blood chemistry, and electrocardiogram |
| Sponsor | Boehringer Ingelheim |
| Notes | Sample size: both studies were powered to detect treatment differences of 0.046 L for FEV_1_ AUC_0–3_ response (SD 0.226 L) with 90% power, and 0.046 L for trough FEV_1_ response (SD 0.225 L) with 92% power  Statistical analysis (for continuous outcomes): restricted maximum likelihood-based mixed model for repeated measurements. |

| **ENERGITO** (NCT01969721) | |
| --- | --- |
| Objective | To assess the effect on lung function of once-daily tiotropium + olodaterol versus twice-daily salmeterol + fluticasone propionate in all participants with GOLD 2 or 3 (moderate to severe) COPD |
| Methods | Complete crossover design (four arms)  Double-blind, double-dummy  Phase IIIb  Multicentre (n=29) and multinational (Europe; 8 countries)  Treatment period (x4): 6 weeks  Washout period: 3 weeks |
| Participants  (elegibility) | Inclusion criteria:   - A diagnosis of COPD - Moderate-to-severe pulmonary impairment (postbronchodilator FEV_1_ ≥30% and <80% of predicted normal) - Postbronchodilator FEV_1_/FVC <70% at screening visit - Age ≥40 years - Current or ex-smoker with a smoking history of >10 pack-years - Able to perform technically acceptable PFT and maintain paper diaries as required - Able to competently inhale medication from the Respimat® inhaler (a metered-dose inhaler) and the Accuhaler®   Key exclusion criteria:   - Significant disease other than COPD - Any COPD exacerbation requiring treatment with antibiotics, systemic steroids, or hospitalization in the past 3 months - Abnormal laboratory tests according to the investigator - History of asthma |
| Participants  (characteristics) | Age (mean; SD): 63.6 (7.6)  Gender (%male): 64.6%  GOLD: 1 (0%), 2 (72.1%), 3 (27.9%), 4 (0%)  COPD diagnosis, mean (SD), years: not reported  % of predicted normal FEV1: 56.4% (11.8)  Current smokers: 44.5%  Smoking history (pack-years): 39.1 |
| Participants  (flow) | Enrolled: 288  Randomized: 229  Analyzed:   - Tiotropium + olodaterol (2.5/5 µg): 93:44% (214/229) - Tiotropium + olodaterol (5/5 µg): 94.32% (216/229) - Salmeterol + fluticasone propionate (50/250 µg): 92.13% (211/229) - Salmeterol + fluticasone propionate (50/500 µg): 94.75% (217/229) |
| Interventions | Experimental:   - Tiotropium (5 µg) + olodaterol (5 µg), via Respimat^®^, once daily - Tiotropium (2.5 µg) + olodaterol (5 µg), via Respimat^®^, once daily   Control:   - Salmeterol (50 µg) + fluticasone propionate (500 µg), via Accuhaler^®^, twice-daily - Salmeterol (50 µg) + fluticasone propionate (250 µg), via Accuhaler^®^, twice-daily   Treatment duration: 6 weeks (each treatment period)  Co-interventions:  Salbutamol as rescue medication use  Short-acting anticholinergic (ipratropium bromide) was used during the screening and washout periods |
| Outcomes | Primary:   - Response (change from baseline) in terms of FEV_1_ AUC_0–12_   Secondary:   - FEV_1_ AUC_0–24_ - FEV_1_ AUC_12–24_ - Peak_0–3_ FEV_1_ response - FEV_1_ response - Adverse events - FVC response - Peak_0–3_ FVC response - FVC AUC_0–24_, FVC AUC_0–12_, and FVC AUC_12–24_ responses - FEV_1_ and FVC measured at all time points after 6 weeks of treatment |
| Sponsor | Boehringer Ingelheim |
| Notes | Efficacy analyses were performed for all participants who received at least one dose of trial medication and had both baseline and post-baseline measurements for the primary end point, defined as the full analysis set  Sample size calculation and assumptions: not reported  Statistical analysis (for continuous outcomes): mixed effects model repeated measures |

| **MORACTO 1 & 2** (NCT01533922 and NCT01533935) | |
| --- | --- |
| Objective | To investigate the effect of 6 weeks treatment with tiotropium + olodaterol fixed dose combination inhalation solution on lung hyperinflation and exercise tolerance in patients with COPD |
| Methods | Randomized, 4-period incomplete cross-over design (5 arms)  Double-blind  Phase III  Multicentre and international   - MORACTO 1 (n=44 centres in Argentina, Australia, Austria, Belgium, Canada, Chile, Germany, Italy, New Zealand, and USA) - MORACTO 2 (n=33 centres in Argentina, Austria, Canada, Germany, Netherlands, Russia, Sweden, and USA)   Treatment period (x5): 6 weeks  Run-in period: 2-6 weeks  Washout period: 3 weeks |
| Participants  (elegibility) | Inclusion criteria:   - Diagnosis of chronic obstructive pulmonary disease - Relatively stable airway obstruction with a post-bronchodilator FEV_1_ <80% of predicted normal and a post-bronchodilator FEV_1_/FVC <70% at Visit 1 - Male or female patients, between 40 and 75 years of age (inclusive) - Current or ex-smokers with a smoking history of more than 10 pack years   Exclusion criteria:   - Significant disease other than COPD - Unstable or life-threatening cardiac arrhythmia - Hospitalization for heart failure or myocardial infarction within the past year - Regular use of daytime oxygen therapy for >1 hour per day - History of asthma - Patients with contraindications to exercise as per European Respiratory Society guidelines |
| Participants  (characteristics) | **MORACTO 1**  Age (mean): 62.2 years  Gender (%male): 72%  GOLD: not reported  **MORACTO 2**  Age (mean): 61.2 years  Gender (%male): 70%  GOLD: not reported |
| Participants  (flow) | **MORACTO 1**  Randomised: 295  Completed: 252 (85%)  **MORACTO 2**  Randomised: 291  Completed: 249 (86%) |
| Interventions | Experimental:   - Tiotropium (5 μg) + olodaterol (5 μg) in a fixed dose combination once daily, via the Respimat® inhaler - Tiotropium (2.5 μg) + olodaterol (5 μg) in a fixed dose combination once daily, via the Respimat® inhaler   Control:   - Tiotropium (5 μg), once daily, via the Respimat® inhaler - Olodaterol (5 μg), once daily, via the Respimat® inhaler - Placebo, once daily, via the Respimat® inhaler   Co-interventions:  Inhaled corticosteroids if taken at baseline  Open-label salbutamol (albuterol) as rescue medication |
| Outcomes | Primary:   - Inspiratory capacity at rest before constant work rate cycle ergometry (CWRCE) to symptom limitation at 75% maximal work capacity - Endurance time during constant work rate cycle ergometry to symptom limitation at 75% work capacity   Secondary:   - Slope of the intensity of breathing discomfort during constant work rate cycle ergometry to symptom limitation at 75% work capacity - FEV_1_ - Adverse events - Post hoc subgroup analyses were performed based on quartiles of post-bronchodilator FEV_1_ percentage predicted normal at baseline |
| Sponsor | Boehringer Ingelheim |
| Notes | Sample size calculation and assumptions: not available  Statistical analysis (for continuous outcomes): mixed effects model repeated measures  Completed but not yet reported as a paper  Results posted in clinicaltrials.gov |

| **OTEMTO 1 & 2** (NCT01964352 and NCT02006732) | |
| --- | --- |
| Objective | To evaluate the effect of tiotropium + olodaterol on lung-function improvement and health related quality of life after 12 weeks of treatment compared to placebo and tiotropium 5 mg in patients with moderate to severe COPD |
| Methods | Parallel design (four arms)  Double-blind  Phase III  Multicentre and multinational   - OTEMTO1: 77 centres in Belgium, Canada, Czech Republic, Denmark, Finland, Germany, South Africa, Spain, United Kingdom, and United States) - OTEMTO2: 78 centres in Australia, Austria, Canada, Germany, Greece, New Zealand, Norway, Slovakia, South Africa, Sweden, and United States)   Follow up (& end point): 12 weeks  Run-in period: 2 weeks |
| Participants  (elegibility) | Inclusion criteria:   - Age: 40 years - Moderate to severe chronic obstructive pulmonary disease (GOLD 2-3) - Post-bronchodilator FEV_1_ ≤30% and <80% of predicted normal - FEV_1_/FVC <70% predicted - Smoking history >10 pack-years   Exclusion criteria:   - History of asthma - Significant disease other than COPD - COPD exacerbation or symptoms of lower respiratory tract infection within the previous 3 months - Unstable or life-threatening cardiac arrhythmia - Hospitalisation for heart failure within the past year - History of myocardial infarction within 1 year of screening - History of life-threatening pulmonary obstruction |
| Participants  (characteristics) | **OTEMTO1**  Age (mean range): 64.7 to 65.1  Gender (male): 56.2% to 62.3%  GOLD: 2 (65.1%), 3 (34.3%)  COPD time since diagnosis: not reported  % of predicted normal FEV1: 54.9 – 56.3% (12.0 – 13.7)  Any pulmonary medication at baseline: 76.5 – 79.7%  Current smokers: 43.1 – 54.7%  Smoking history (pack-years): not reported  **OTEMTO2**  Age (mean range): 64.0 to 65.2  Gender (male): 57.9% to 65.8%  GOLD: 2 (63.5%), 3 (36.1%)  COPD time since diagnosis: not reported  % of predicted normal FEV1: 54.3 – 55.9% (12.0 – 13.4)  Any pulmonary medication at baseline: 70.3 – 77.8%  Current smokers: 44.6 – 47.0%  Smoking history (pack-years): not reported |
| Participants  (flow) | **OTEMTO1**  Screened: 1054  Randomised: 814   - Tiotropium + olodaterol 2.5/5 μg: 202 - Tiotropium + olodaterol 5/5 μg: 204 - Tiotropium 5 μg: 204 - Placebo: 204   Completed: 92.26% (751/814)  **OTEMTO2**  Screened: 1107  Randomised: 809   - Tiotropium + olodaterol 2.5/5 μg: 202 - Tiotropium + olodaterol 5/5 μg: 202 - Tiotropium 5 μg: 203 - Placebo: 202   Completed: 94.43% (764/809) |
| Interventions | Experimental:   - Tiotropium (2.5 μg) plus olodaterol (5 μg), once daily, Respimat^®^ inhaler - Tiotropium (5 μg) plus olodaterol (5 μg), once daily, Respimat^®^ inhaler   Control:   - Placebo, once daily, Respimat^®^ inhaler - Tiotropium (5 μg), once daily, Respimat^®^ inhaler   Treatment duration: 12 weeks  Co-interventions:  Inhaled corticosteroid therapy (if they were on a stable dose for 6 weeks prior to screening)  Open-label salbutamol as rescue medication for use throughout the study |
| Outcomes | Primary:   - St. George Respiratory Questionnaire - FEV_1_ AUC_0-3_ response (change from baseline) - FEV_1_ response   Secondary:   - Mahler Transition Dyspnoea Index focal score - FVC and FVC AUC_0-3_ responses - All adverse events - Serious adverse events |
| Sponsor | Boehringer Ingelheim |
| Notes | Sample size: both studies were powered (90%) to detect a difference of 0.073 L in FEV_1_ AUC_0-3_ and trough FEV_1_ (assuming standard deviations of 0.226 L for FEV_1_ AUC_0-3_ and 0.225 L for trough FEV_1_), and 3.2 units in SGRQ total score (assuming a standard deviation of 14 units)  The primary analyses were intent-to-treat analyses, performed on the full analysis set, defined as all patients who received at least one dose of study medication and had baseline and at least one post-baseline measurement for any of the primary end points  Statistical analysis (for continuous outcomes): mixed effects model repeated measures |

| **TONADO 1 & 2** (NCT01431274 and NCT01431287) | |
| --- | --- |
| Objective | To assess the efficacy and safety of once-daily treatment with orally inhaled tiotropium+olodaterol FDC 5/5 μg or 2.5/5 μg delivered via the Respimat compared with their individual mono-components in patients with moderate to very severe COPD (GOLD stage 2–4) over 52 weeks |
| Methods | Parallel design (5 arms)  Double-blind  Phase III  Multicentre and international (25 countries)  Follow up: 52 weeks  End point: 24 weeks |
| Participants  (elegibility) | Inclusion criteria:   - Male or female patients aged ≥40 years - History of moderate to very severe chronic obstructive pulmonary disease (GOLD stage 2–4) - Post-bronchodilator forced expiratory volume_1_ <80% of predicted normal; post-bronchodilator FEV_1_/FVC <70% - Current or ex-smokers with a smoking history of >10 pack–years   Key exclusion criteria:   - Significant disease other than severe COPD - History of asthma - Respiratory tract infection or COPD exacerbation occurring ≤6 weeks prior to screening - Regular use of daytime oxygen if patients were unable to abstain during clinic visits |
| Participants  (characteristics) | Age (mean, SD): 63.8 – 64.2 (7.8 – 8.7)  Gender (male): 71.2% - 73.6%  GOLD: 2 (50.1%), 3 (38.5%), 4 (10.8%)  COPD time since diagnosis: not reported  % of predicted normal FEV1: 49.3 – 50.3% (14.9 – 15.7)  Current smokers: 35.8 – 38.9%  Smoking history (pack-years): not reported |
| Participants  (flow) | Enrrolled: 6837  Randomised: 5163   - Tiotropium + olodaterol (FDC) Respimat 5/5 µg: 1029 - Tiotropium + olodaterol (FDC) Respimat 2.5/5 µg: 1030 - Olodaterol Respimat 5 µg: 1038 - Tiotropium Respimat 5 µg: 1033 - Tiotropium Respimat 2.5 µg: 1032   Completed: 84.40% (4358/5163) |
| Interventions | Experimental:   1. Tiotropium + olodaterol fixed-dose combination Respimat 5/5 µg once daily 2. Tiotropium + olodaterol fixed-dose combination Respimat 2.5/5 µg once daily   Control:   1. Olodaterol Respimat 5 µg once daily 2. Tiotropium Respimat 5 µg once daily 3. Tiotropium Respimat 2.5 µg once daily   Treatment duration: 52 weeks  Co-interventions:  Inhaled corticosteroids as required  Salbutamol/albuterol metered-dose inhaler (100 μg per actuation) as rescue medication to be used as necessary at any point during the trial |
| Outcomes | Primary (* after 24 weeks of treatment):   - FEV_1_ AUC_0–3_ response - Trough FEV_1_ response (change from baseline; mean of the values of 1 h and 10 min prior to the first dose of study medication) - St George’s Respiratory Questionnaire (SGRQ) total score (pre-specified combined analysis of data from both studies)   Secondary:   - Mahler Transition Dyspnoea Index (TDI) focal score (pre-specified combined analysis of data from both studies) - FEV_1_ AUC_0-3_ response on day 1, day 85, and day 365 - Trough FEV_1_ response on day 15, day 43, day 85, day 169, and day 365 - FVC AUC_0–3_ response on day 1, day 85, day 169, and day 365 - Trough FVC response on day 15, day 43, day 85, day 170, and day 365 - FEV_1_ AUC_0–12_ and FEV_1_ AUC_0-24_ response in a 12-h pulmonary function test (PFT) sub-set of patients - FVC AUC_0–12_ and FVC AUC_0-24_ response in a 12-h pulmonary function test (PFT) sub-set of patients - SGRQ total score on day 365 - Mahler TDI focal score on day 43, day 85, and day 365 - Adverse events |
| Sponsor | Boehringer Ingelheim |
| Notes | Sample size calculation and assumptions: not reported  Statistical analysis: not reported |

| **VIVACITO** (NCT01559116) | |
| --- | --- |
| Objective | To demonstrate the 24-h lung function profile and effects on lung volume of tiotropium + olodaterol FDC 2.5/5 μg and 5/5 μg compared to placebo and mono-components after 6 weeks in patients with moderate to very severe chronic obstructive pulmonary disease (GOLD 2-4) |
| Methods | Incomplete cross-over design (6 arms; patients were randomised to receive four of six treatments)  Double-blind  Phase III  Multicentre (n=29) and international (7 countries: Belgium, Canada, Denmark, Germany, Hungary, The Netherlands and the USA)  Duration of treatment (x4): 6 weeks  Washout period: 3 weeks  Run-in: 2-6 weeks |
| Participants  (elegibility) | Inclusion criteria:   1. Age ≥40 years 2. Smoking history of 10 pack-years 3. Relatively stable airway obstruction with a post-bronchodilator FEV_1_ <80% of predicted normal and FEV_1_/FVC <70% of predicted normal   Exclusion criteria:   1. History of asthma or significant disease other than chronic obstructive pulmonary disease 2. Unstable or life-threatening cardiac arrhythmia 3. Hospitalisation for heart failure within the past year, a history of myocardial infarction within 1 year of screening 4. History of life-threatening pulmonary obstruction |
| Participants  (characteristics) | Gender (male): 58.9%  Age (mean; SD): 61.1 (7.7)  Current smokers: 62.6%  GOLD: 2 (63.5%), 3 (34.2%), 4 (2.3%)  COPD time since diagnosis: not reported  % of predicted normal FEV1: 54.0% (13.0)  Current smokers: 62.6%  Smoking history (pack-years): not reported |
| Participants  (flow) | Enrolled: 259  Randomised: 219  Completed (range): 94.2% to 99.3% |
| Interventions | Experimental:   - Tiotropium + olodaterol fixed dose combination 2.5/5 μg via the Respimat^®^ inhaler - Tiotropium + olodaterol fixed dose combination 5/5 μg via the Respimat^®^ inhaler   Control:   - Placebo, via the Respimat^®^ inhaler - Olodaterol 5 μg, via the Respimat^®^ inhaler - Tiotropium 2.5 μg, via the Respimat^®^ inhaler - Tiotropium 5 μg, via the Respimat^®^ inhaler   Treatment duration (x4 of 6): 6 weeks  Co interventions:  Inhaled corticosteroids during treatment periods (if taken as maintenance treatment at study entry)  Short-acting anticholinergics during the screening and washout periods, had to be stopped 8 h before pulmonary function test at the first visit of the next treatment period  Open label salbutamol as rescue medication to be used at baseline and during screening, treatment, washout and follow-up periods |
| Outcomes | Primary:   - FEV_1_ AUC_0-24_ response   Secondary:   - FEV_1_ AUC_0-12_ and FEV_1_ AUC_12-24_ - Lung-volume parameters measured using body plethysmography (subset of patients) - Maximum FEV_1_ value obtained in the first 3 h after dosing (peak _0-3_ FEV_1_) and trough FEV_1_ response, and FVC AUC_0-24_, FVC AUC_0-12_ and FVC AUC_12-24_ responses - Incidence of adverse event |
| Sponsor | Boehringer Ingelheim |
| Notes | Sample size: the study was powered to detect a difference of 60 mL in FEV_1_ AUC_0-24_ with 90% power  The full analysis set was defined as any patient who had taken at least one dose of study medication and had any period baseline and any evaluable post-dose data for the primary endpoint  Statistical analysis (for continuous outcomes): restricted maximum likelihood-based mixed effects model with repeated measures |
