# Supplementary figures and images for: Efficacy and safety of tiotropium and olodaterol in COPD: a systematic review and meta-analysis
Source: Respir Res. 2017 Nov 25;18:196. doi: 10.1186/s12931-017-0683-x (PMC5702233; doi:10.1186/s12931-017-0683-x)

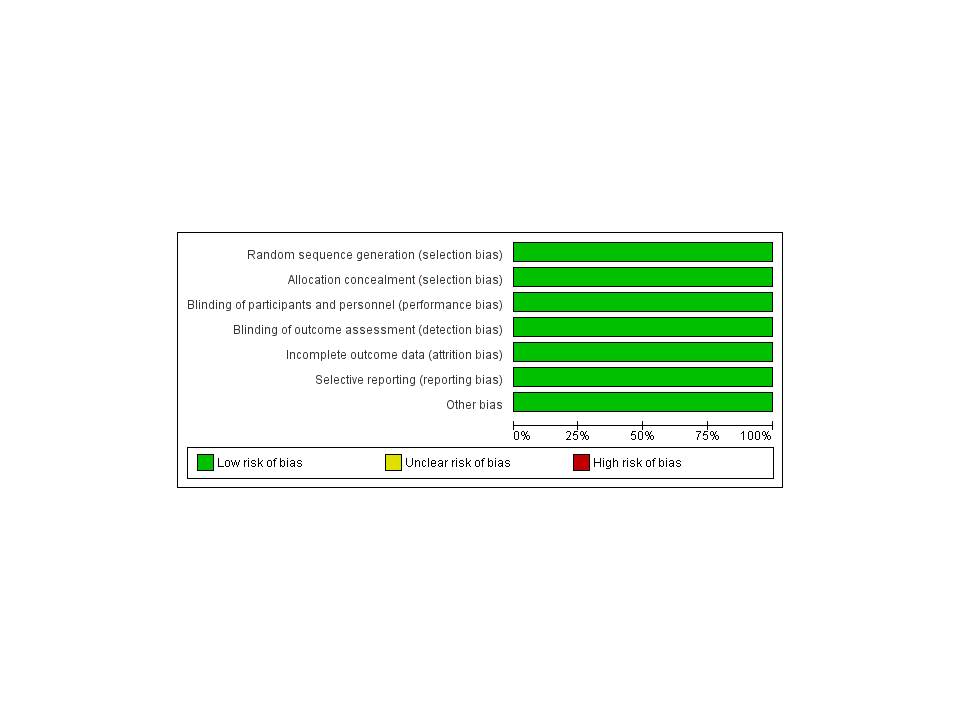

Supplement: Supplementary file 3 — Risk of bias graph. (JPEG 40 kb) [file 12931_2017_683_MOESM3_ESM.jpg]

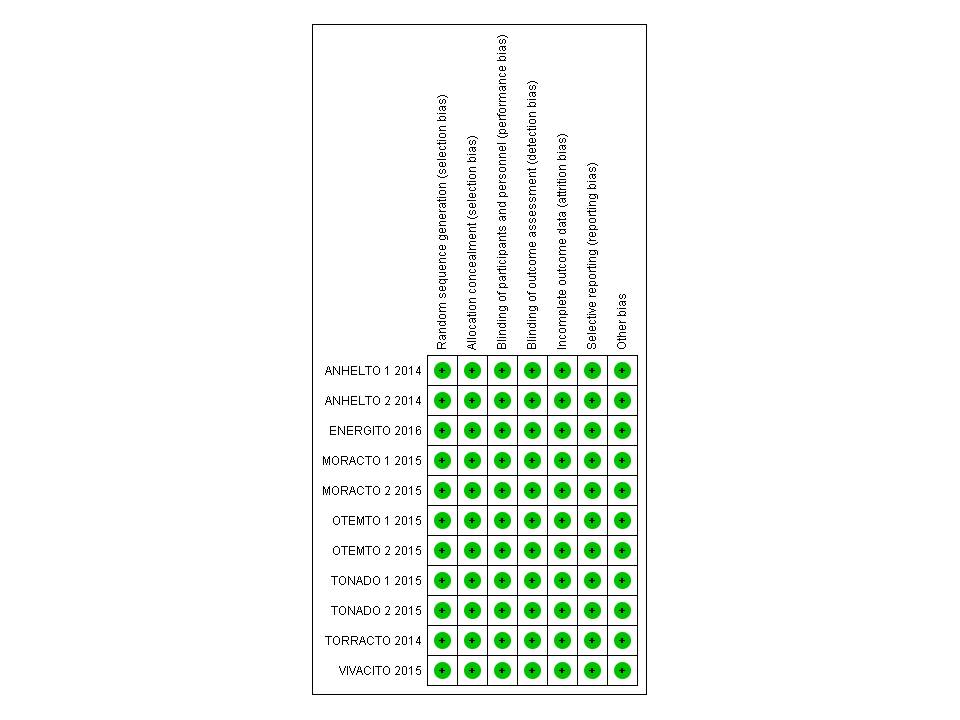

Supplement: Supplementary file 4 — Risk of bias summary. (JPEG 60 kb) [file 12931_2017_683_MOESM4_ESM.jpg]
